# Effects of insulin, human placental lactogen and human growth hormone of DNA synthesis in organ cultures of benign human breast tumours.

**DOI:** 10.1038/bjc.1978.196

**Published:** 1978-08

**Authors:** C. W. Welsch, S. E. Dombroske, M. J. McManus

## Abstract

Nineteen benign human breast tumours, from 19 premenopausal patients, were processed into slices and each tumour was individually cultured for 2 days in Medium 199. The effects of bovine insulin (5.0 microgram/ml) human placental lactogen (10.0 microgram/ml) and human growth hormone (10.0 microgram/ml) on 3H-thymidine incorporation into DNA were determined on the cultured tumour slices. Insulin and human placental lactogen significantly (P less than 0.01) increased the mean incorporation of [3H]TdR into DNA, whereas human growth hormone was ineffective. These results provide evidence that insulin and human placental lactogen, but not human growth hormone, may be important factors in the aetiology of benign human breast tumours.


*Br. J. Cancer* (1978) **38**, 258

### EFFECTS OF INSULIN, HUMAN PLACENTAL LACTOGEN AND HUMAN GROWTH HORMONE ON DNA SYNTHESIS IN ORGAN CULTURES OF BENIGN HUMAN BREAST TUMOURS*

C. W. WELSCH, S. E. DOMBROSKE and M. J. McMANUS

*From the Department of Anatomy, Michigan State University, East Lansing, Michigan* 48824, *USA*

Received 22 May 1978 Accepted 30 May 1978

**Summary.—Nineteen benign human breast tumours, from 19 premenopausal patients, were processed into slices and each tumour was individually cultured for 2 days in Medium 199. The effects of bovine insulin (5·0 μg/ml) human placental lactogen (10·0 μg/ml) and human growth hormone (10·0 μg/ml) on $^3$H-thymidine incorporation into DNA were determined on the cultured tumour slices. Insulin and human placental lactogen significantly ($P < 0{\cdot}01$) increased the mean incorporation of [$^3$H]TdR into DNA, whereas human growth hormone was ineffective. These results provide evidence that insulin and human placental lactogen, but not human growth hormone, may be important factors in the aetiology of benign human breast tumours.**

In a recent report, we provided evidence that both insulin and human placental lactogen (HPL) were mitogenic to the ductal epithelium in benign human breast tumours (Welsch and McManus, 1977). The mitogenic activity of these peptides was assessed *in vitro* by measuring DNA synthesis of 2-day organ cultures of freshly biopsied benign breast dysplasias. Both peptides increased: (1) the incorporation of [$^3$H]TdR into chemically extracted DNA, (2) the [$^3$H]TdR autoradiographic labelling index and (3) the mitotic index of these dysplasias, suggesting that one or both of these peptides may play a role in the aetiology of this disease.

Human growth hormone (HGH) is a pituitary peptide, chemically, immunologically and physiologically similar to HPL (Forsyth, 1974; Bewley and Li, 1974; Handwerger and Sherwood, 1974). The pituitary peptide has been reported to have lactogenic and mammary growth-promoting activities in lower animals (Forsyth, 1974; Handwerger and Sherwood, 1974) but its effect on growth and differentiation of human breast tissues is not known. Because of the close structural similarities of HGH and HPL, it was of interest to determine whether or not HGH has mitogenic activities toward benign human breast tumours, as has been reported for HPL (Welsch and McManus, 1977).

### MATERIALS AND METHODS

Nineteen human benign breast-tumour biopsy specimens, obtained from 19 premenopausal patients, were placed in a chilled holding medium and returned to the laboratory within one hour. The biopsy specimens were immediately and carefully trimmed of adipose tissue while immersed in the holding medium. All tissue preparations were performed in a laminar-flow hood under aseptic conditions.

*Preparation of slices for organ culture.*—Slices of biopsy specimens were prepared with the aid of a Stadie-Riggs tissue slicer. Each biopsy specimen provided 5–15 large slices ranging from 10–15 mm in diameter and 0·1–0·3 mm in thickness. Each slice was processed by a series of halvings with a surgical blade (*i.e.*, each half was successively halved

* Supported by American Cancer Society research grant BC-220C.

until the slices measured ~ 1 × 1 mm). The small slices (1 × 1 mm) were pooled and placed in 10 × 35 mm Falcon disposable Petri dishes, 10 slices/dish. Each Petri dish contained 2·0 ml of the culture medium.

Each biopsy specimen was divided into either 3 or 4 groups (*i.e.*, a control and either 2 or 3 experimental groups). Each group (controls and experimental) had 9 small Petri dishes containing a total of 90 small slices. The small Petri dishes were placed in a covered water-saturated larger Falcon disposable Petri dish (15 × 100 mm), 3 small dishes per larger dish. The Petri dishes were then placed in a small gassing chamber and housed in an incubator at 37 °C. The chambers were continuously infused with gas (95% $O_2$—5% $CO_2$) during the culture period. All biopsy specimens were individually cultured; slices from different specimens were never combined. The large number of randomly selected small slices per group provides reasonable assurance that an equal quantity of epithelium is distributed among the groups at the onset of culture.

The culture medium used in these studies was Medium 199, modified Earle's salts obtained from Grand Island Biological Co., Grand Island, NY. The hormones used in this study, and their concentrations in the culture media were: bovine pancreas insulin (California Biochemical Corp., La Jolla, CA, 22·5 i.u./mg) (5·0 μg/ml); human placental lactogen (HPL, Nutritional Biochemical Corp., Cleveland, OH) (10·0 μg/ml); human growth hormone (HGH, NIH-HS2160E) (10·0 μg/ml) and HGH (California Biochemical Corp, La Jolla, CA) 10·0 μg/ml. All media contained gentamicin (Schering Corp., Kenilworth, NJ) (50·0 μg/ml). After all additions, the media were passed through a Millipore filter (0·45 μm), added to the Petri dishes, and the entire culture assembly was frozen ( − 20°C) until the biopsy specimens were brought to the laboratory.

At the end of the 2nd day of culture, 4 h before termination, sterile methyl-[$^3$H]TdR (New England Nuclear, Boston, MA; 2·0 Ci/mmol) was added to the culture medium, at 1·0 μCi/ml. Termination of the cultures was designed to facilitate quick removal of the small slices from the media (in order to obtain a wet weight for each group) and then storage in 0·9% NaCl at −20°C until DNA extraction and analysis.

*DNA extraction and analysis of cultured slices.*—For DNA extraction and analysis, the tissues from each group were ground in 0·9% NaCl solution with a Willems Polytron homogenizer. An equal volume of 20% trichloracetic acid was added to the homogenate the resulting precipitate was centrifuged (3000 *g*) and washed twice with 10% trichloracetic acid. The precipitate was then washed twice in sodium acetate in methanol and in chloroform-methanol, once in 100% ethanol and once in 100% ethyl ether, in that order, to remove lipid and $H_2O$. In all the foregoing procedures, the preparations were kept constantly cold. The defatted-dehydrated extract was placed in a ventilated fume hood (12–18 h), then in a vacuum desiccator (24 h) and was subsequently weighed.

The defatted-dehydrated extract was digested (3 h at 37°C) with repeated stirrings in 0·3N KOH. The preparation was cooled, precipitated with cold 10% perchloric acid, centrifuged (3000 *g*) and washed twice. The precipitate was then incubated for 30 min with constant stirring in hot (70°C) 5% perchloric acid in which the DNA was soluble. This preparation was cooled, centrifuged (3000 *g*) and washed twice with cold 5% perchloric acid. The supernatant was collected for DNA and [$^3$H]TdR analysis. DNA content was quantitatively determined (in duplicate) by the diphenylamine-colorimetric method of Burton (1956). Calf thymus DNA (Sigma Chemical Co., St. Louis, MO) was used as a standard. The [$^3$H]TdR content was determined by pipetting aliquots (in triplicate) of the supernatant into modified Bray's scintillation fluid. The samples were counted in a Beckman LS-100c liquid scintillation counter with a counting efficiency of 51%. The results were expressed as ct/min [$^3$H]TdR per μg DNA. Significance of differences between mean ct/min/μg DNA values of each group was analysed by the *t* test for paired observations.

### RESULTS

The addition of insulin or HPL to culture medium containing explants of benign human breast tumours significantly ($P < 0{\cdot}01$) increased the mean incorporation of [$^3$H]TdR into DNA (Figs. 1 and 2). The addition of a commercially prepared HGH (HGH-2) or that prepared

FIG. 1.—Effects of insulin and human growth hormone (HGH) on [$^3$H]TdR incorporation into DNA of 2-day organ cultures of 10 benign human breast tumours. Control *vs* insulin, $P < 0{\cdot}01$. Control *vs* HGH, not significant.

FIG. 2.—Effects of human placental lactogen (HPL) and human placental lactogen plus human growth hormone (HGH) on [$^3$H]TdR incorporation into DNA of 2-day organ cultures of 9 benign human breast tumours. Control *vs* HPL or HPL + HGH, $P < 0{\cdot}01$. HPL *vs* HPL + HGH, not significant.

by the U.S. National Institutes of Health (HGH-1) did not significantly influence the mean incorporation of the isotope into DNA (Fig. 1) nor did the pituitary peptide, in combination with HPL, enhance the mitogenic action of the placental peptide (Fig. 2). All 19 breast-tumour explants actively incorporated [$^3$H]TdR into DNA during organ culture, to a level comparable to that previously reported by our laboratory (Welsch and McManus, 1977). Nearly all the benign tumours were classified histologically as fibrocystic disease with varying degrees of adenosis and ductal hyperplasia. No correlation between histopathological diagnosis and response to hormones was seen.

### DISCUSSION

Unlike malignant human breast tissue, normal or benign human breast tissue can be consistently and effectively maintained in short-term organ culture (Welsch *et al.*, 1976; Welsch and McManus, 1977). This provides a unique opportunity to study a variety of factors influential in the development, growth and differentiation of the human breast. DNA synthesis of tissue *in vitro* can be reliably assessed by the addition of a pulse label of [$^3$H]TdR 4 h before termination of culture. We have previously reported (Welsch and McManus, 1977) that this method of quantitating DNA synthesis provides data which *directly* correlates with [$^3$H]TdR labelling indices and mitotic figure analysis (*i.e.*, when [$^3$H]TdR incorporation into chemically extracted DNA is increased, invariably the [$^3$H]TdR labelling index and mitotic figure index are similarly increased). Thus the procedure for assessing DNA synthesis used in this study has the advantage of being time saving yet still retains the precision for measuring mitotic activity.

Although we have previously reported that insulin and HPL are stimulatory to DNA synthesis of organ cultures of benign human breast tumours (Welsch and McManus, 1977) neither our laboratory, nor others, has previously investigated whether or not HGH is mitogenic to these tissues. Whereas we could again demonstrate a stimulatory effect of insulin and HPL on these dysplasias, purified HGH from 2 independent sources did not show a significant stimulatory effect.

Both HPL and HGH are single-chain peptides of 190 amino-acid residues with 2 interchain disulphide bonds (Bewley and Li, 1974). The amino-acid compositions of the 2 hormones are very similar, the only major differences being in the number of methionine, histidine and proline residues. The 2 hormones are identical at 163 of the 190 residues, accounting for 86% homology in the amino-acid sequences. Despite this structural similarity, the somatotrophic and lactogenic activities of these peptides in lower animals are not always parallel. In comparing the lactogenic activities of HPL and HGH, the lactogenic potency of HPL is essentially identical to HGH when measured by the pigeon-crop assay, the pseudopregnant rabbit mammary intraductal assay or the mid-pregnant mouse mammary organ culture assay (Forsyth, 1974; Handwerger and Sherwood, 1974). The pigeon assay uses mucosa-cell proliferation as an end point, the rabbit assay uses milk production as an end point and the mouse assay uses alveolar-cell proliferation and milk production as end points. In general, both peptides have slight but significant stimulatory effects in the pigeon crop assay and substantial stimulatory effects in the intraductal and organ-culture assays. Thus both peptides in *lower animals* appear to be not only lactogenic but mammotrophic as well. In comparing the general somatotrophic activities of the 2 peptides, it is apparent that the 2 hormones have very different growth-promoting potencies, despite their structural similarities. Li (1972) has reported that HPL has 13% of the activity of HGH in the rodent tibia test and it is generally agreed that the general growth-promoting potency of HPL in man is minimal (McGarry and Beck, 1972).

Despite the close chemical and physiological similarities of HPL and HGH, there appears to be a marked difference between them in their ability to stimulate DNA synthesis of benign human breast tumours. This observation is important, because it may provide insight into the structural entity of HPL prerequisite for human mammotrophic activities, a site that may not be analogous to that for lower animals for which both HPL and HGH appear to be mammotrophic.

HPL has been reported to stimulate growth of precancerous mammary-gland dysplasias in mice (Yanai and Nagasawa, 1973). Whether or not human benign breast dysplasias are precancerous remains to be determined, although patients bearing such dysplasias are in an increased breast-cancer risk group (MacMahon and Cole, 1973). Prolactin, a pituitary peptide structurally similar to HPL, is an important hormone in murine mammary tumorigenesis, whereas purfied growth hormone has not been definitively shown to be mammary oncogenic in lower animals (Welsch and Nagasawa, 1977). The results presented in this study provide evidence that HPL (but not HGH) may be an important hormonal factor in the etiology of benign disease of the human breast.

### REFERENCES

Bewley, T. A. & Li, C. H. (1974) Structural similarities between human pituitary growth hormone human somatomammotropin, and ovine pituitary growth and lactogenic hormones. In *Lactogenic Hormones, Fetal Nutrition, and Lactation*. Eds J. B. Josimovich, M. Reynolds and E. Cobo. New York: John Wiley & Sons. p. 19.

Burton, K. (1956) A study of the conditions and mechanism of the diphenylamine reaction for the colorimetric estimation of deoxyribonucleic acid. *Biochem. J.*, **62**, 315.

Forsyth, I. A. (1974) The comparative study of placental lactogenic hormones: a review. In. *Lactogenic Hormones, Fetal Nutrition, and Lactation*. Eds J. B. Josimovich, M. Reynolds and E. Cobo. New York: John Wiley & Sons. p. 49.

Handwerger, S. & Sherwood, L. M. (1974) Comparison of the structure and lactogenic activity of human placental lactogen and human growth hormone. In *Lactogenic Hormones, Fetal Nutrition, and Lactation*. Eds J. B. Josimovich, M. Reynolds and E. Cobo. New York: John Wiley & Sons. p. 33.

Li, C. H. (1972) Recent knowledge of the chemistry of lactogenic hormones. In *Lactogenic Hormones*. Eds G. E. W. Wolstenholme and J. Knight. London: Churchill-Livingstone. p. 7.

MacMahon, B. & Cole, P. (1973) Etiology of human breast cancer. A review. *J. Natl. Cancer Inst.*, **50**, 21.

McGarry, E. E. & Beck, J. C. (1972) Biological effects of non-primate prolactin and human placental lactogen. In *Lactogenic Hormones*. Eds G. E. W. Wolstenholme and J. Knight. London: Churchill-Livingstone. p. 361.

WELSCH, C. W., CALAF DE ITURRI, G. & BRENNAN, M. J. (1976) DNA synthesis of human, mouse, and rat mammary carcinomas *in vitro*. Influence of insulin and prolactin. *Cancer*, **38**, 1272.

WELSCH, C. W. & McMANUS, M. J. (1977) Stimulation of DNA synthesis by human placental lactogen or insulin in organ cultures of benign human breast tumors. *Cancer Res.*, **37**, 2257.

WELSCH, C. W. & NAGASAWA, H. (1977) Prolactin and murine mammary tumorigenesis. A review. *Cancer Res.*, **37**, 951.

YANAI, R. & NAGASAWA, H. (1973) Enhancement by human placental lactogen of mammary hyperplastic nodules in ovariectomized mice. *Cancer Res.*, **33**, 1642.


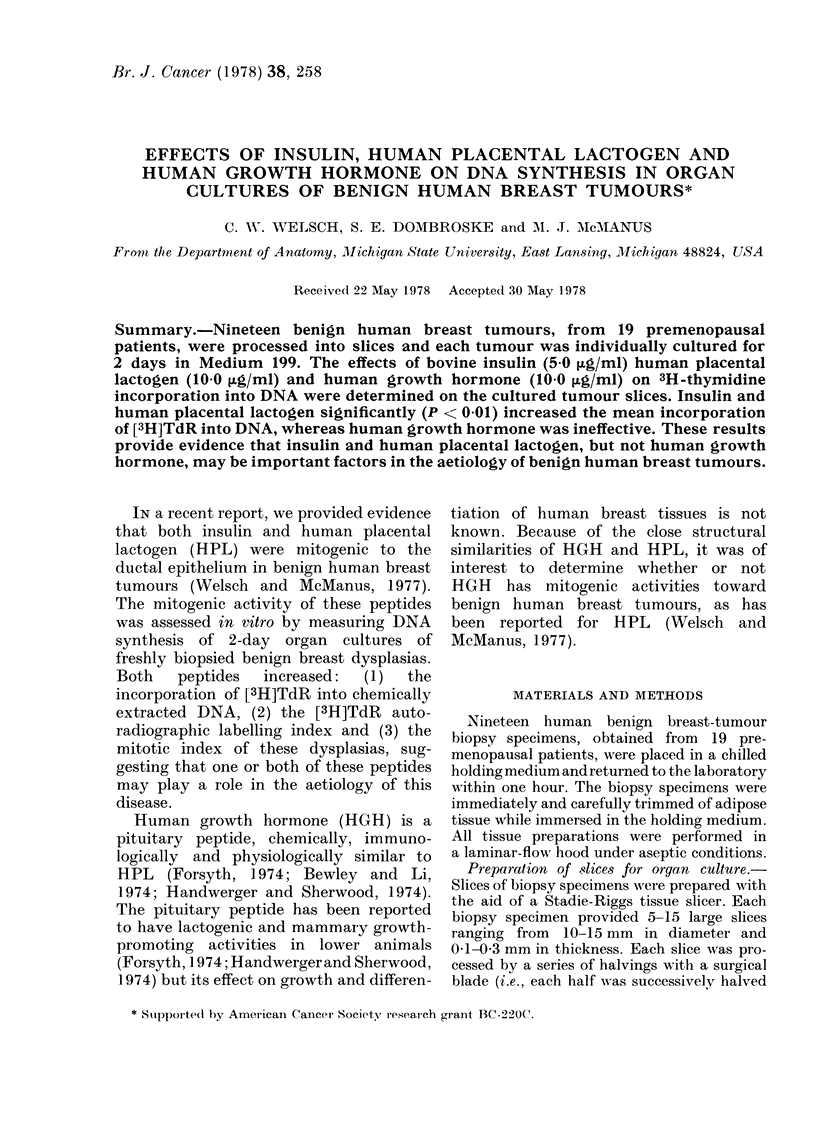

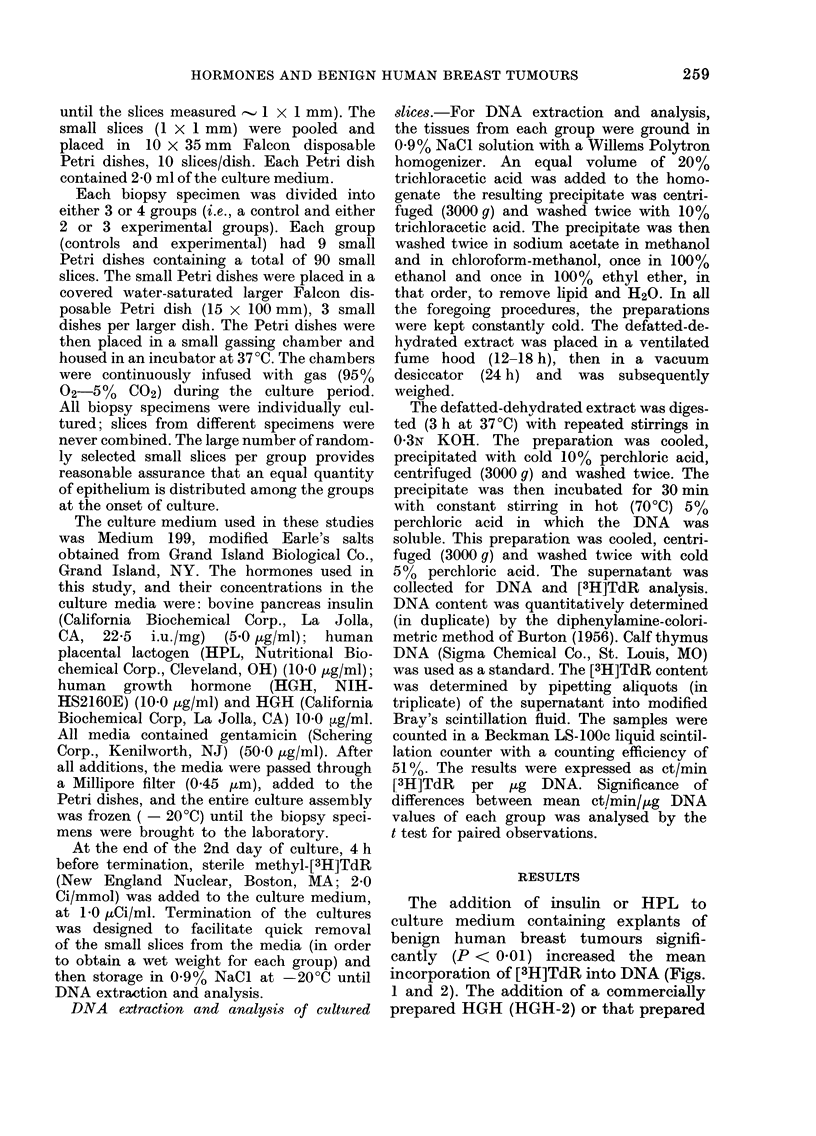

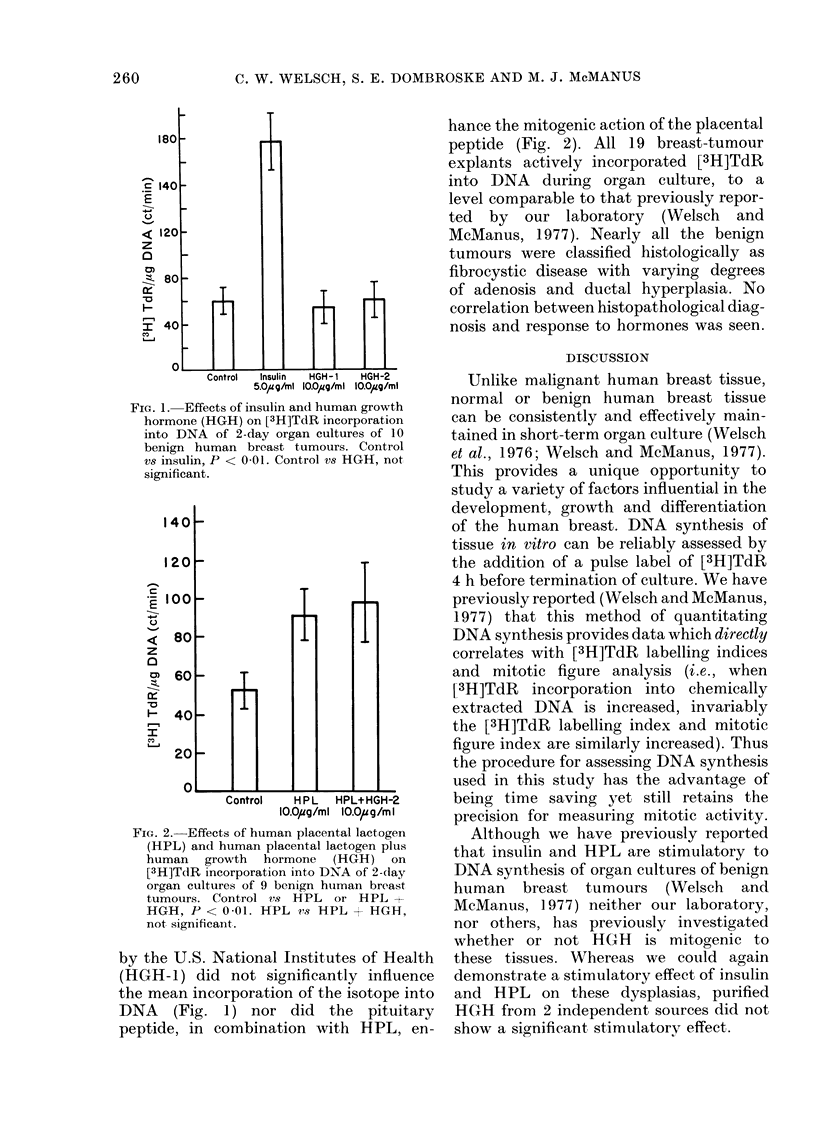

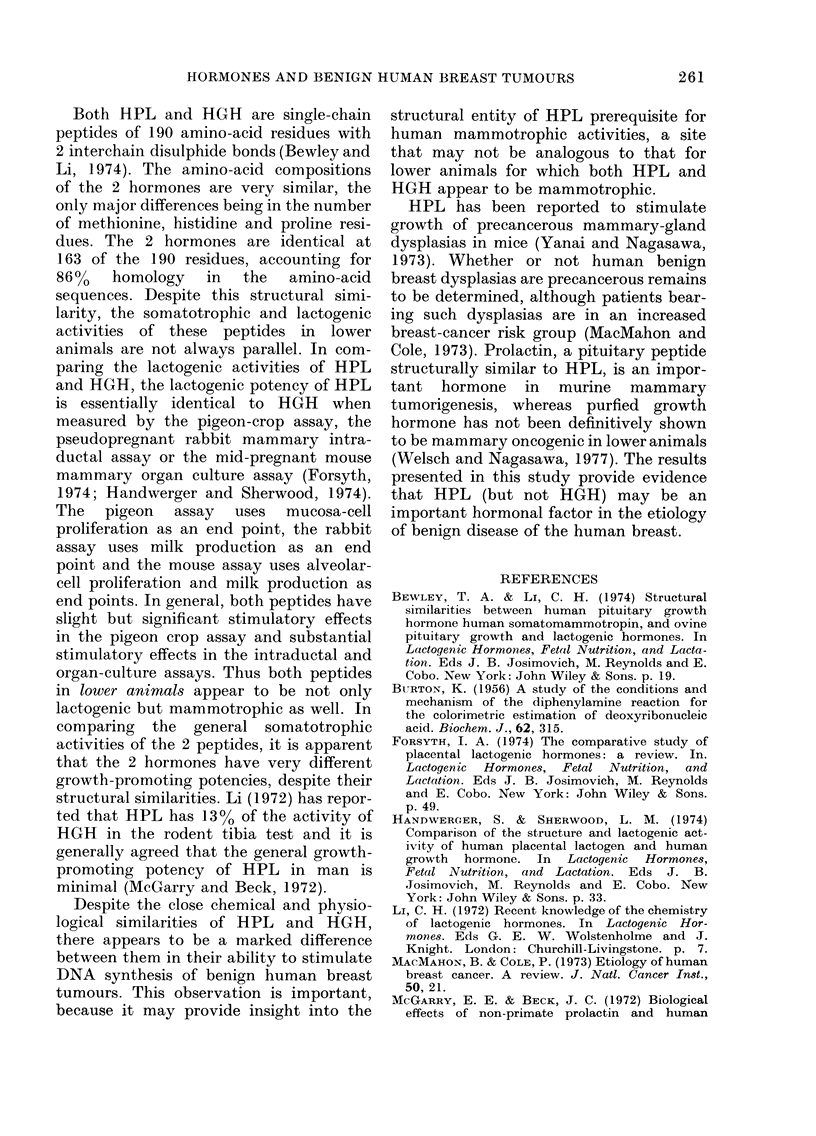

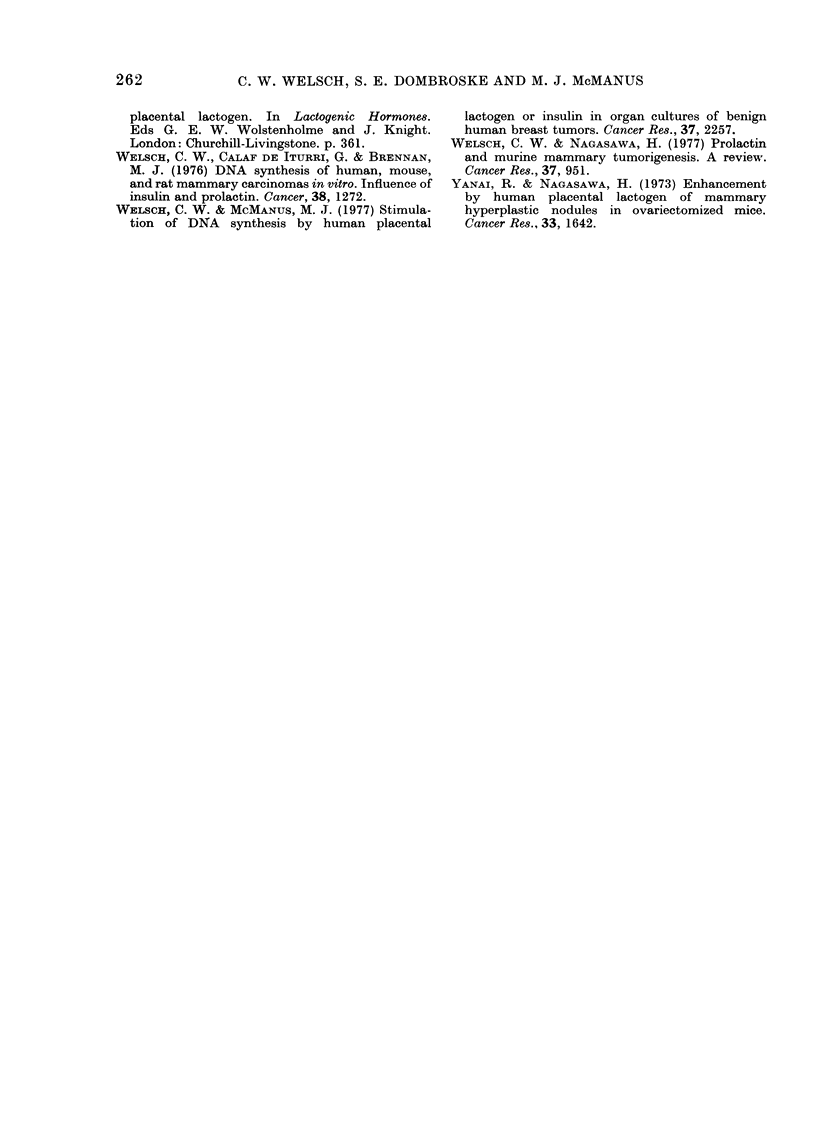


## References

[OCR_00478] BURTON K. (1956). A study of the conditions and mechanism of the diphenylamine reaction for the colorimetric estimation of deoxyribonucleic acid.. Biochem J.

[OCR_00506] MacMahon B., Cole P., Brown J. (1973). Etiology of human breast cancer: a review.. J Natl Cancer Inst.

[OCR_00521] Welsch C. W., Iturri G. C., Brennan M. J. (1976). DNA synthesis of human, mouse, and rat mammary carcinomas in vitro: influence of insulin and prolactin.. Cancer.

[OCR_00527] Welsch C. W., McManus M. J. (1977). Stimulation of DNA synthesis by human placental lactogen or insulin in organ cultures of benign human breast tumors.. Cancer Res.

[OCR_00534] Welsch C. W., Nagasawa H. (1977). Prolactin and murine mammary tumorigenesis: a review.. Cancer Res.

[OCR_00539] Yanai R., Nagasawa H. (1973). Enhancement by human placental lactogen of mammary hyperplastic nodules in ovariectomized mice.. Cancer Res.

